# Reversible Jump MCMC for Deghosting in MSPSR Systems †

**DOI:** 10.3390/s21144815

**Published:** 2021-07-14

**Authors:** Pavel Kulmon

**Affiliations:** Department of Applied Informatics, Czech Technical University in Prague, 166 29 Prague, Czech Republic; pavel.kulmon@fsv.cvut.cz

**Keywords:** FM, radar, MSPSR, Bayesian inference, deghosting, MCMC, reversible jump

## Abstract

This paper deals with bistatic track association and deghosting in the classical frequency modulation (FM)-based multi-static primary surveillance radar (MSPSR). The main contribution of this paper is a novel algorithm for bistatic track association and deghosting. The proposed algorithm is based on a hierarchical model which uses the Indian buffet process (IBP) as the prior probability distribution for the association matrix. The inference of the association matrix is then performed using the classical reversible jump Markov chain Monte Carlo (RJMCMC) algorithm with the usage of a custom set of the moves proposed by the sampler. A detailed description of the moves together with the underlying theory and the whole model is provided. Using the simulated data, the algorithm is compared with the two alternative ones and the results show the significantly better performance of the proposed algorithm in such a simulated setup. The simulated data are also used for the analysis of the properties of Markov chains produced by the sampler, such as the convergence or the posterior distribution. At the end of the paper, further research on the proposed method is outlined.

## 1. Introduction

Passive location systems are widely used in both civil and military applications. Due to their low hardware cost (no transmitters needed), they offer reliable solutions for covert operations and offer the advantage of fast deployment. Among the passive sensors, the multi-static primary surveillance radars (MSPSRs) with bistatic geometry offer unique advantages. Foremost, no target transmission is necessary and due to the bistatic geometry, it is difficult to design such an aircraft surface, which would prevent the reflected signal being spread in the receiver direction. The frequency modulation (FM)-based MSPSRs are used either separately or together with single-frequency network (SFN) systems which suffer from transmitter uncertainty.

The usual MSPSR data processing architecture, which is also suggested, e.g., in [1] is as follows. After signal reception and processing, the detection process is performed. This process produces primary data which is usually comprised of bistatic range and bistatic velocity. There are systems where the angle of arrival or angle of elevation is also available, but this requires a special kind of receiving antenna and therefore the angles are not available in every MSPSR system. Tracking in bistatic coordinates (which is especially possible in FM-based systems where the transmitter uncertainty is eliminated) reduces the concentration of false measurements and also increases the precision of target bistatic coordinates [2]. After the validation phase, bistatic tracks from different receiver–transmitter pairs are associated and form groups of at least three bistatic tracks from distinct bistatic spaces. From each group of associated bistatic tracks, a new Cartesian target is initiated [3]. The bistatic track association process usually includes the deghosting part, which is a method of false association hypothesis elimination. The deghosting can be either explicitly performed after forming all of the available hypotheses, or implicitly being part of the association process.

There are many solutions for the deghosting problem available in the literature [4,5,6,7,8]. A deghosting algorithm based on the evaluation of all possible bistatic track pair combinations with modified clustering is available in [9]. This algorithm is primarily focused on SFN systems; however, it can be as effectively used for an FM-based system. A different approach is chosen in [6] which explores the usage of residual error as a deghosting criterion, however, only in the case of 2D localisation. In [4], another method based on multiple association algorithms and so-called super targets formation was developed. This method can also be used for both SFN- and FM-based MSPSR systems. The multidimensional assignment problem is solved in [10] using the Lagrangian relaxation technique with an application to target localisation in the network of direction finders.

The majority of the deghosting algorithms available in the literature are based on the evaluation of as many association hypotheses as possible. These hypotheses are then reduced using some testing criterion or some association heuristic, such as the linearised joint probabilistic data association (JPDA) to optimise the results. However, there are also deghosting approaches which use the Bayesian approach. The Markov chain Monte Carlo data association (MCMCDA) [11] relies on the specifically designed path in the association space and defines the prior probability distribution on this association. This prior probability distribution can be influenced using design parameters such as the probability of detection or false target concentration, which are hard to estimate in real scenarios. Other Bayesian approaches such as probability hypothesis density (PHD) [12] or probabilistic multiple hypothesis track (PMHT) [13] use likelihood functions but do not formulate any prior or they use it only in the context of false measurements. In this paper, we also use the Bayesian approach for MSPSR deghosting, however, we define the bistatic track association as a Bayesian inference problem and develop the Markov chain Monte Carlo (MCMC) sampler to solve this discrete-continuous inference problem and determine the most likely solution. To do this, we need to establish prior probability distribution for the modified association matrix which is introduced in the subsequent sections together with the proposed sampler. This probabilistic model forms a novel deghosting algorithm for the MSPSR applications. We then compare the results of our new deghosting method with results obtained using two alternative deghosting algorithms. The comparison is performed using simulated data with known truth. We illustrate the superior performance of our algorithm over the two others and also discuss the achieved results. Moreover, the simulated data are used in the sampler to produce multiple long runs of the chain which is later analysed in order to assess its convergence. As this is an extended version of [14], the detailed convergence assessment and the rest of the detailed analysis is the additional contribution. Illustration of chain iterations in the Cartesian space and the target identification space are provided. At the end of the results section, the analysis of the discovered association hypotheses posterior distribution is presented. At the end of the paper, the results and methodology are discussed and the paper is concluded with the outline of further research.

## 2. Materials and Methods

### 2.1. Assignment Problem

The assignment problem in target tracking is concerned with obtaining the assignment matrix $A=\left(a_{ij}\right)$ where $a_{ij}\in \left\{0,1\right\}$ between two sets of entities V,W. If $a_{ij}=1$, then we decide on assignment between entity i∈V and j∈W. Usually, we put some constraints on the form of the assignment matrix, such as the uniqueness of the assignment. Such a constraint can be formulated using the following conditions:

(1) $$\begin{array}{c} \sum_{i}a_{ij}\leq 1,\\ \sum_{j}a_{ij}\leq 1, \end{array}$$

which ensures that entities from one set are not shared between entities from the other set. However, there are situations where such a constraint is not appropriate, such as assignment between bistatic tracks and Cartesian targets in MSPSR target initiation/deghosting. If we are concerned with only one bistatic space, then in the simple case, we can put the constraints (1) on the assignment matrix. In the simple case, we consider using a hard assignment decision that assigns bistatic detections to tracks and thus ensures probabilistic independence between two different Cartesian targets. For the rest of the paper, we will assume that this condition be fulfilled. However, one bistatic space is not enough to estimate the target position and velocity in 3D Cartesian space. Therefore, we usually need at least three bistatic spaces so that the target initiation/deghosting stage can be performed. Such a task usually consists of an evaluation of all possible combinations of bistatic tracks from different bistatic spaces of various cardinality (e.g., two or three tracks) and the statistical testing of resulting Cartesian target positions. We suggest keeping the association matrix and establish new constraints, which would only be concerned with a submatrix representing bistatic tracks from the same bistatic space. From the global point of view, the first condition in (1) no longer holds; on the contrary, we encourage the bistatic tracks from different bistatic spaces to share the assignment with the same Cartesian target (as we previously mentioned, a minimum of three tracks is necessary, however, we want the target to be tracked in every bistatic space available). We can express the previous description of MSPSR assignment matrix A as

(2) $$A=\left(\begin{array}{c} A^{1}\\ A^{2}\\ \vdots \\ A^{n_{BS}} \end{array}\right),$$

where the upper index of submatrices denotes the index of a particular bistatic space and $n_{BS}$ is the overall number of bistatic spaces available. For any *k*, we apply conditions (1) on the submatrix $A^{k}$. From (2), it is obvious that the more tracks there are that share assignment with the same Cartesian target (i.e., the more ones there are in the same columns while keeping conditions (1)), the fewer columns are necessary to express the assignment (i.e., the less Cartesian targets will be initiated). This is a desirable property because the problem with many of the deghosting algorithms is the number of possible track combinations which provide feasible results (i.e., an estimate of the Cartesian position with small residual error). One of the ways to incorporate this property into the solution of the target initiation/deghosting process is the enumeration of all possible associations of all the cardinalities from $n_{BS}$ to 3. Some of the associations can be dismissed based on the residual error, and the other can be omitted because its tracks are already part of the feasible assignment of a larger cardinality, which is preferred. However, in extreme cases, the combinatorial complexity of this computation is unbearable. Another option is to incorporate the desired properties of the assignment matrix into its prior and evaluate not only the probabilities of bistatic track assignments based on the residual error of Cartesian position, but also the probability of the assignment matrix concerning this prior.

### 2.2. Indian Buffet Process

Let us recall the properties of the assignment matrix. We want the initiated target (without loss of generality represented by columns) to be shared by multiple bistatic tracks from distinct bistatic spaces, and in the hard assignment setting, each bistatic track to be assigned to at most one initiated target. The number of targets is usually unknown or can be only roughly estimated. Therefore, another property of a prior for the assignment matrix is that it should be flexible enough concerning the number of targets. This requirement is often used in nonparametric Bayesian modelling [15] as the structure of observed data may not be completely known. This is similar to the case of MSPSR target initiation/deghosting since we do not know which targets produced detections of a given bistatic track. In the environment of nonparametric Bayesian statistics, the prior distribution for binary matrices with a finite number of rows (the observed data) and a potentially infinite number of columns (the way the unknown number of columns is modelled) is defined using a simple generative process called Indian buffet process (IBP). The name of the generative process is derived from the metaphor used to describe the process. A description of the process together with its statistical properties can be found in [16]. The probability distribution of matrices, under which the order of targets is exchangeable, requires us to define classes of equivalence between assignment matrices in such a way, that two matrices from the same class represent the same underlying structure in the data. The exchangeability is also a desired property in MSPSR target initiation/deghosting because the order of targets is not important, as long as they are represented by the right set of bistatic tracks. The equivalence classes are defined using the $lof\left(•\right)$ function [17] which represents the columns of the assignment matrix as binary numbers (assignment represents a true value) with the first row being the most significant bit before sorting them as integers. For any association matrix A, we denote its $lof\left(•\right)$ equivalence class as $\left[A\right]$. With assignment matrices represented by equivalence class, we can define the probability distribution for different classes. There are two versions of probability distributions for $lof\left(•\right)$ classes of binary matrices which differ in the number of parameters. The first version [15] only depends on parameter α. This parameter controls shared assignments between different bistatic tracks (in our application, with the restriction on bistatic space submatrices from (1)) and also the expected number of initiated targets. Since in the IBP for each row we sample from Poisson distribution to add new columns, in the limit case, the number of columns is Nα where *N* is the row count of the association matrix (i.e., the number of bistatic tracks over all of the bistatic spaces). The second probability distribution uses two parameters—α and β. In this representation, α represents the average number of assignments for one bistatic track and β controls how the assignments to one particular target are shared between bistatic tracks (the smaller β is, the more the assignments are shared). For the rest of the paper, we will use the two-parameter representation, since it allows better modelling of assignment matrices in MSPSR target initiation/deghosting.

For any assignment matrix A, the probability of its $lof\left(•\right)$ equivalence class is [16]:

(3) $$P\left(\left[A\right]\right)=\frac{{\left(\alpha \beta \right)}^{K_{+}}}{\prod_{h\leq 1}K_{h}!}\exp\left(-{\bar{K}}_{+}\right)\prod_{k=1}^{K_{+}}B\left(m_{k},N-m_{k}+\beta \right),$$

where $K_{+}$ is the number of assignment matrix columns with at least one 1 (i.e., targets with at least one piece of associated data), $m_{k}$ is the number of associated data for column *k*, $K_{h}$ is the number of identical columns and ${\bar{K}}_{+}$ is the expected number of columns for binary matrix distribution with parameters α and β and is equal to:

(4) $${\bar{K}}_{+}=\alpha \sum_{i=1}^{N}\frac{\beta }{\beta -i+1}.$$

**Figure 1:**
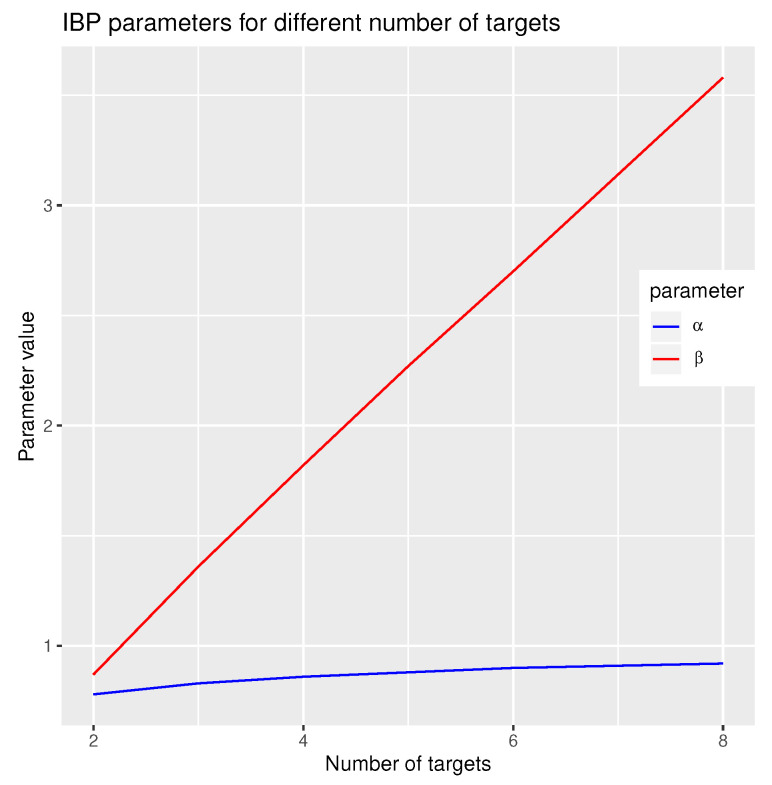
Indian buffet process (IBP) prior parameter values for which the ideal assignment matrix has maximum probability.

In Figure 1, the different IBP parameter values are visualised for a different number of targets in four bistatic spaces with an ideal assignment matrix. We can see, as the number of targets grows, the relative number of shared bistatic tracks decreases and hence the β parameters value increases.

### 2.3. Sampler

In the subsequent sections, we describe the sampler used to solve the inference problem. This custom sampler is based on the design of a set of moves in the Markov chain that (a) reversibility (explained later) is assured and (b) the set together with respective proposal distribution allows us to efficiently explore the whole parametric space. In general, the sampler is based on the reversible jump Markov chain Monte Carlo (RJMCMC) theory, first introduced in [18]. Since its introduction, this method was extensively studied and used in many different applications, e.g., [19,20,21,22].

### 2.4. RJMCMC

The reversible jump Markov chain Monte Carlo (RJMCMC) [18] is an extension of the MCMC class of algorithms for generating samples from probability distributions. In particular, RJMCMC extends the Metropolis–Hastings (M-H) [23] algorithm for the case, when “the number of unknown parameters is one of the unknown parameters” [22]. Such applications are called transdimensional since during the inference of the parameters, we are required to sometimes change the dimension of the parametric space. Such a description is certainly valid for the case of the deghosting problem in the MSPSR system, as it was presented in the Introduction. The core task is to decide which bistatic tracks belong to each other and thus effectively decide upon the number of valid combinations, i.e., the number of modelled targets. From the purely combinatoric perspective, even with the lower limit of three bistatic tracks per target and the upper limit of at most one bistatic track from one bistatic space, the number of options is usually very large. The geometrical perspective eliminates many of these options, however, such a process is computationally exhaustive.

The complications with reduced geometry (i.e., bistatic versus Cartesian space) together with no information about the possible target locations are one of the main reasons to use the RJMCMC methodology. The classical model selection methodology would not be applicable here because enumerating all of the possible models is very expensive. The comparison of different models would then bring even more difficulties such as how to ensure the combinations of as many bistatic tracks to be preferred. During the review of this paper, the use of the approximate Bayesian computation (ABC) [24] was proposed. While we agree that it could be used to solve the problem at hand, the design of an appropriate summary statistic would be in our opinion as complex a problem as the design of the RJMCMC sampler is.

We can think about different numbers of targets as different models. Each target is parametrised by its position $p\in R^{3}$ and velocity $v\in R^{3}$ which are coupled in to the state vector $x\in R^{6}$. Thus, different models (different numbers of targets) also differ in the number of parameters. For the model *k*, we denote by $n_{k}$ the number of parameters of the model, which is equal to $n_{k}=6n_{k}^{t}$ where $n_{k}^{t}$ is the number of targets in the model. We denote the point in the parametric space $x=\left(k,\theta_{k}\right)$ where *k* is a label of the model and:

(5) $$\theta_{k}=\left(x_{1}^{k},x_{2}^{k},\cdots ,x_{n_{k}^{t}}^{k}\right).$$

The whole parametric space over which the problem is defined is then given by subspaces $L_{k}=\left\{k\right\}\times R^{n_{k}}$ for one model, and thus, by taking the union $L={⋃}_{k}L_{k}$, we obtain all possible parametrisations over the set of all model indices. One of the assumptions behind the sampling procedure is the decomposition of the joint density, given the data Z (without specification of what they consist of):

(6) $$p\left(k,\theta_{k},Z\right)=p\left(k\right)p\left(\theta_{k}|k\right)p\left(Z|k,\theta_{k}\right),$$

which follows from the idea that the probability density of parameters is easy to specify once the dimension is given, but otherwise very hard to formulate. In the same way, the posterior density factorisation is given by [18]

(7) $$p\left(k,\theta_{k}|Z\right)=p\left(k|Z\right)p\left(\theta_{k}|k,Z\right).$$

Without getting too extensively into the theory of RJMCMC (more details on which can be found by an interested reader in the references of this section), we only emphasise that in this paper, it is only used as the extension of the classical M-H algorithm. Let us denote by $x=\left(k,\theta_{k}\right)$ the current state of the Markov chain. Using some (later specified) proposal distribution $q\left(x,x^{\prime }\right)$, we propose a new state $x^{\prime }$ which is accepted as the new state that the chain moves to with the probability:

(8) $$\rho \left(x^{\prime },x\right)=\min\left\{1,\frac{\pi \left(x^{\prime }\right)q\left(x^{\prime },x\right)}{\pi \left(x\right)q\left(x,x^{\prime }\right)}\right\},$$

where π is the target distribution of the chain, i.e., the distribution we would like the samples to be sampled from. Please note that (8) is a plain M-H acceptance ratio if for *x* and $x^{\prime }$ the model dimensions do not differ. The acceptance ratio is based on the notion of reversibility of the constructed chain (which ensures the existence of the stationary distribution) and also its ergodicity (which ensures the uniqueness of the stationary distribution) [25]. In RJMCMC [20], these assumptions are considered and by the measure-theoretic approach, these assumptions are extended to much more general state spaces such as L. The equilibrium equation is formulated using the transition kernel K of the Markov chain:

(9) $$\int_{L}\pi \left(dx\right)K\left(x,dx^{\prime }\right)=\pi \left(dx^{\prime }\right).$$

In the same manner, the reversibility for two subsets $B,B^{\prime }\subset L$ can be expressed as

(10) $$\int_{B}\pi \left(dx\right)K\left(x,B^{\prime }\right)=\int_{B^{\prime }}\pi \left(dx^{\prime }\right)K\left(x^{\prime },B\right).$$

This formulation guarantees the desired Markov chain to be constructed even for transdimensional models. For a more detailed description, please refer to [18,19,20,22,26].

### 2.5. Parametric Space

Let us describe the properties of the parametric space before we describe the full model and the sampler. We now assume that a fixed realisation of A, the association matrix, is available. We denote the number of columns of A by *K*, which also represents the current number of targets in the evaluated model. From the previous section, we can observe that $n_{k}=K$; however, we need to drop the model index for the sake of notation simplicity as the association matrix and thus also the model is fixed. We assume the second condition in (1) to hold that the association must be consistent. For any *i*, *j* the $a_{ij}=1$ means that the *j*-th target is associated with (and therefore its position is given by) *i*-th bistatic measurement. We assume the measurement errors in bistatic space to come from a multivariate normal distribution and thus the measurements are sufficiently described by state vector $z_{i}\in R^{2}$ (in our application. only the bistatic range and bistatic velocity are measured) and covariance matrix $Z_{i}\in R^{2\times 2}$. The transformation function $h_{i}:R^{6}\to R^{2}$ is specific for each measurement *i* and is given by the positions of receivers and transmitters of the respective bistatic space that the measurement comes from. Note that for the set of measurements associated with one target, all of the transformation functions need to be independent. The likelihood function of the *j*-th target position $x_{j}$ concerning the *i*-th measurement can then be written as

(11) $$p\left(x_{j}|z_{i},Z_{i}\right)=N\left(h\left(x_{j}\right)|z_{i},Z_{i}\right),$$

where $N\left(x|\mu ,\Sigma \right)$ denotes the multivariate normal distribution with the mean vector μ and covariance matrix Σ, evaluated at the point x. The choice of this likelihood function is natural. The detections in the bistatic space (the bistatic tracks are created using these detections) have their covariance matrix estimated from the signal properties in their neighbourhood. In this case, the actual measurement errors should be normally distributed. Then, if we assume a linear (or almost linear) target movement model in the bistatic space, normality is preserved. Since the measurements in different bistatic spaces are independent, the likelihood function of the target position $x_{j}$ conditioned by the whole association matrix is given by

(12) $$p\left(x_{j}|A,Z\right)=\prod_{a_{ij}=1}N\left(h_{i}\left(x_{j}\right)|z_{i},Z_{i}\right),$$

where by Z, we denote the set of all measurements. Therefore, for a fixed realisation of A, the target estimates $\hat{\theta }={\left\{{\hat{x}}_{j}\right\}}_{j=1}^{K}$ can be found using, e.g., the method of maximum likelihood, by

(13) $${\hat{x}}_{j}=\underset{x_{j}}{\mathrm{argmax}}\;p\left(x_{j}|A,Z\right),$$

and this maximisation can be naturally performed independently for each target in the model. Such an approach offers two advantages. First, there are numerous methods specifically designed to solve the problem (13). A straightforward solution for the case of normal likelihood cannot be used due to the nonlinearity of the function h. Therefore, the solution has to be found using some iterative maximisation algorithm. Starting point ${\hat{x}}_{j}^{0}=\left({\hat{p}}_{j}^{0},{\hat{v}}_{j}^{0}\right)$ can be found using three bistatic positions [27]

(14) $${\hat{p}}_{j}^{0}=a+bR_{t},$$

where:

(15) $$\begin{array}{cc} a= & {\left(S^{T}S\right)}^{-1}S^{T}s,\\ b= & {\left(S^{T}S\right)}^{-1}S^{T}r, \end{array}$$

and $R_{t}$ is the distance between the target and central receiver station, r is the vector of bistatic positions, S is the row matrix of transmitter position coordinates and $s=\frac{1}{2}\left[\left(S^{o2}\cdot i\right)-r^{o2}\right]$, $i\in R^{3\times 1}$ is their vector. In the equation for s, the operator $B^{o2}$ denotes the Hadamard second power of the matrix B. Details of this approach together with its derivation and means to compute the velocity estimate can also be found in [2,27]. Once the initial estimate is obtained, the final estimate ${\hat{x}}_{j}$ can be found through the convex optimisation [28] or using the Newton method [29]. The second advantage of this approach is the efficient marginalisation of the target parameters from the sampled distribution. Once the association matrix is sampled, the probability distribution of target parameters is obtained using the maximum-likelihood method based on the bistatic tracks assigned. This makes the development of an efficient sampler much easier since we are only concerned with binary assignment variables. A similar approach was used in [30] where the transition probabilities in the hidden Markov model were estimated using the backward–forward algorithm.

### 2.6. Model

**Figure 2:**
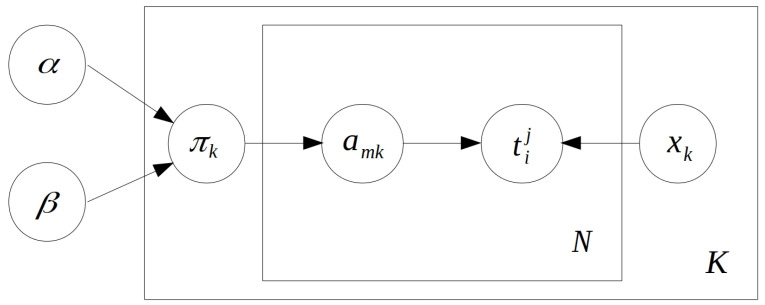
Graphical visualisation of the bistatic tracks association model. Parameters α and β define the Beta distributed (used in the definition of the IBP) hidden variables $\pi_{k}$ for a matrix of *K* columns. Association matrix entries $a_{mk}$ define the association between bistatic tracks $t_{j}^{i}$ and targets $x_{k}$. By *N*, we denote the overall number of bistatic tracks available.

In the next section, we describe the sampling procedure which we use to perform moves across the whole parametric space. Here, we simply summarise the graphical model based on the prior probability distribution for the association matrix (3), which is formulated using the IBP, and the probability distribution of the data observed, conditioned on target parameters. The probability model arising from the definition of IBP [17] is given by

(16) $$\begin{array}{cc} \pi_{k}|\alpha ,\beta ∼ & \mathrm{Beta}\left(\frac{\alpha \beta }{K},\beta \right),\\ a_{mk}|\pi_{k}∼ & \mathrm{Bernoulli}\left(\pi_{k}\right), \end{array}$$

where $\pi_{k}$ is the latent variable, connected for all of the matrix columns through the IBP parameters α and β. By $a_{mk}$, we denote the *m*-th element of *k*-th column of the association matrix to prevent confusion with an indexation of bistatic tracks. To complete the probabilistic model, we denote by $t_{j}^{i}$ the bistatic data of *i*-th track in *j*-th bistatic space. Using the notation of the previous section, we can write:

(17) $$t_{j}^{i}|a_{m(i,j)k}=1,x_{k}∼N\left(h_{j}\left(x_{k}\right),Z_{m(i,j)}\right),$$

and here we use $m\left(i,j\right):N^{2}\to N$ as a transformation from a pair of indices (of bistatic track *i* in bistatic space *j*) to a linear index. This is only a formality with no influence on the model. Having such a transformation simply allows us to differentiate between the transformation functions of different bistatic spaces. The overall structure of the model in graphical form is available in Figure 2.

### 2.7. Basic Moves

The description of how we estimate the target parameters once the number of targets is given is presented in Section 2.5. Here, we analyse the options for the sampler to jump between the association matrices, either with the same number of columns or transdimensionally.

There are two general classes of transdimensional moves available in the literature on RJMCMC. The first class is the birth-and-death (BD) [18] class of moves and the second class is split-and-merge moves (SM) [21]. The BD class was introduced together with the new theory of RJMCMC [18], extensively analysed in the context of other MCMC methods [19] and also appears in newer papers concerning the RJMCMC theory [22,30,31]. The BD move assumes that if the currently assumed dimension is *K* (e.g., representing the number of changepoints in time-series or the number of targets), we can propose either a decrease in the dimension (K−1) or its increase (K+1). In the applications available in the literature, such a process usually involves a type of parameter transformation as the *K* vectors of parameters are dependent. This is not true for the case of bistatic track association since we still preserve the second condition of (1) and single targets in the model are therefore independent (and so are its parameters). It is also possible to propose the BD move at random, i.e., in the case of the birth move, we can propose a random 6D vector of the new target parameters. There is no harm in the association consistency since the new target does not possess any bistatic tracks. However, we argue that concerning the high-dimensional space of target occurrence, this move would not be efficient. There is also a problem with the likelihood evaluation for a target without data, and we would need to propose some arbitrary likelihood function. In the case of a death move, similar problems arise. Randomly, we can propose some track to be deleted. However, we would end up with a set of unassigned bistatic tracks. Such an operation would break the consistency requirement of the association procedure. With this analysis, we decided not to implement BD moves in our sampler, despite the BD type of moves being considered as basic in the RJMCMC literature.

**Figure 3:**
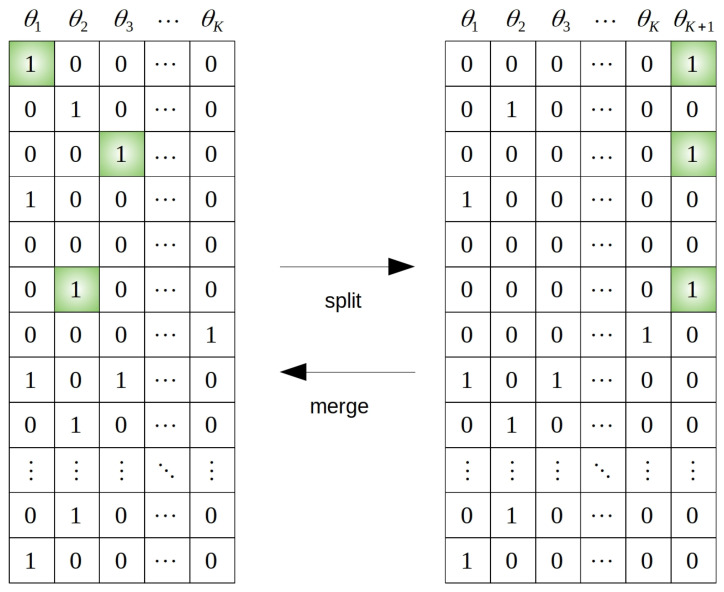
Matrix visualisation of the reversible split-and-merge move. Here, we mainly focus on the target-to-track association and omit the differences between bistatic spaces (although we assume the association to be consistent). Columns are labelled by $\theta_{j}$, which denotes the *j*-th target rather than the target state vector $x_{j}$ or its estimate ${\hat{x}}_{j}$.

The second class of RJMCMC moves, called split-and-merge, was introduced in [32] and later extended in [21]. Again, the moves are proposed as a reversible pair so that the detailed balance condition (10) is still fulfilled. We start with the description of the split move. Let us suppose that K>1 and we generate random S≥3. By *S*, we denote the number of bistatic tracks we randomly select in existing targets (while preserving the association consistency) and we propose to establish a new target using this set of tracks. The move also contains the operation of removing selected tracks from their respective targets. The randomness of *S* is not necessary and its value should also be selected concerning the computational effort. Initiation from S=3 tracks is also possible and advisable with respect to the closed-form solution (14). In addition, selecting smaller sets of bistatic tracks rather than some large ones increases the acceptance ratio because the probability of set contamination by an inconsistent track is lower. Overall, the deterministic setting $S\in \left\{1,3,4\right\}$ proved itself to be a good strategy. The case when S=1 is the special case of an SM move which serves to sample clutter targets. Such targets do not exist in the observed space; however, from the chain perspective, they serve as storage of inconsistent tracks (inconsistent with other targets). Another good modification is to prefer selecting tracks from those targets, which are either underdetermined, or the data likelihood (i.e., the column likelihood) is very low (hence the target is probably very inconsistent). The merge move is similarly designed. We pick one target at random. For every one of these target bistatic tracks, we (randomly) select some other target to which we would like to assign the bistatic track. This way, the pair is reversible since with every move, there is a way to return to the original state. An example of the SM reversible pair is available in Figure 3. We will now describe the reversible pair more formally from the point of the MH acceptance ratio. We denote by A the current association matrix and by $A^{\prime }$ the association matrix which would be the result of the proposed move (i.e., a matrix with K+1 columns in the case of the split move and K−1 columns in the case of the merge move). Similarly, we denote by θ the current set of target state vectors and by $\theta^{\prime }$ the set of target state vectors of the proposed move. The acceptance ratio is then given by

(18) $$\rho \left(\left(A^{\prime },\theta^{\prime }\right),\left(A,\theta \right)\right)=\min\left\{1,\frac{p\left(Z|A^{\prime },\theta^{\prime }\right)p\left(A^{\prime }|\alpha ,\beta \right)q_{m}\left(A^{\prime },A\right)}{p\left(Z|A,\theta \right)p\left(A|\alpha ,\beta \right)q_{m}\left(A,A^{\prime }\right)}\right\},$$

where the probability $p\left(A^{\prime }|\alpha ,\beta \right)$ is given by the IBP prior and is evaluated for the whole *lof* equivalence class. The proposal distribution $q_{m}\left(A^{\prime },A\right)$ was described in the previous paragraph and the specific value depends on the number of targets and bistatic tracks available. Note that we use the ratio given by proposal distributions $q_{m}\left(A^{\prime },A\right)/q_{m}\left(A,A^{\prime }\right)$ of the specific SM move instead of the distribution of all possible moves [21]. This significantly simplifies the computations. This transdimensional move changes the parameters of some of the existing targets involved in the random selection. However, the parameters are solely given by the data contained in the bistatic tracks, the likelihood of which is evaluated as the first term in the acceptance ratio (18). With respect to this observation, we assume the Jacobian usually contained in the RJMCMC ratio [18] to be the identity matrix. The data likelihood in (18) arises from (11) as

(19) $$p\left(Z|A,\theta \right)=\prod_{a_{mk}=1}N\left(z_{m}|h_{m}\left({\hat{x}}_{k}\right),Z_{m}\right).$$

Concerning the already presented target parameters’ marginalisation, this quantity always represents the maximum likelihood value (both concerning the target state and the bistatic track data). Note that the acceptance ratio for proposing new targets can be high, especially concerning the potential number of false ideal intersections. This would lead to the constant creation of new targets which, due to the prior probability distribution of the association matrix, would then be eventually merged with previous estimates of the same target from a different set of data. To prevent this, we decided to put a small penalisation $\lambda_{j}$ on the data probability of new targets. The term (19) then becomes:

(20) $$p\left(Z|A,\theta \right)=\prod_{a_{mk}=1}N\left(z_{m}|h_{m}\left({\hat{x}}_{k}\right),Z_{m}\right)\exp\left(\lambda_{j}\right),$$

where:

(21) $$\lambda_{j}=\left\{\begin{array}{cc} {\bar{\lambda }}_{j} & \sum_{i=1}^{N}a_{ij}<4,\\ 0 & otherwise. \end{array}\right.$$

The penalisation constant ${\bar{\lambda }}_{j}$ is chosen in such a way that the accepted proposals do not cause the fragmentation of the larger internally consistent group into smaller subsets of measurements. Our experiments show the importance of such a penalisation.

A different approach was taken in the case of the second type of moves we used. We call them reassignment (R). This move is not transdimensional because it does not change the number of targets in the model. Hence, the regular MH acceptance ratio applies. From the current assignment matrix, A, we randomly select one entry $a_{ij}$ and propose its change to a complement value ${\bar{a}}_{ij}$. Since the association consistency has to be preserved, we also select some $a_{il}$, which, in the current state of the chain, has the value $a_{il}={\bar{a}}_{ij}$. The complement value is defined as

(22) $${\bar{a}}_{ij}=\left\{\begin{array}{cc} 1 & a_{ij}=0,\\ 0 & a_{ij}=1. \end{array}\right.$$

Note that for the first case of (22), there is only one *l* possessing the complement value, while for the second case, any l≠j is available. This, together with the actual values of *N* and *K*, defines the transition distributions $q_{m}$ of this move. There is also the option to propose the assignment change in a deterministic way. For every $a_{ij}$, there is either K−1 or 1 options to change the assignment. The number of entry selections is NK and therefore the complexity of such a deterministic proposal would be $O\left(NK^{2}\right)$. Note, however, that the proposals are not independent and for the reversibility to be preserved, different conditions need to be developed. The probability of the assignment matrix having the value $a_{ij}=1$ follows from the two-parametric IBP prior to the distribution [33] and is given by

(23) $$p\left(a_{ij}=1|A,\alpha ,\beta \right)=\frac{m^{-ij}}{N-m^{-ij}-\beta },$$

where $m^{-ij}$ is computed as the number of tracks assigned to the target *j* excluding the track *i*. As in the case of SM move, we denote here by $A^{\prime }$ the new assignment matrix with changed value $a_{ij}^{\prime }$ and $a_{il}^{\prime }$ at their respective positions. The acceptance ratio in general would be (with the modified proposal distribution $q_{m}$) the same as in (18). However, there is a large space for the optimisation of computational cost. For both directions of this MCMC move, only the *i*-th and *l*-th columns of the association matrix are changed. Due to this, only ${\hat{x}}_{j}$ and ${\hat{x}}_{l}$ change value and therefore change the data likelihood. Moreover, the probability with respect to the prior is given by (23) rather than (3) which also significantly simplifies the computation. We also exploited the independence of single columns concerning the data likelihood and therefore we can write the acceptance ratio in the following form:

(24) $$\rho \left(\left(A^{\prime },\theta^{\prime }\right),\left(A,\theta \right)\right)=\min\left(1,\frac{\prod_{k\in \left\{j,l\right\}}\left[\prod_{i,a_{ik}^{\prime }}N\left(z_{i}|h\left({\hat{x}}_{k}^{\prime }\right),Z_{i}\right)\right]p\left(a_{ij}^{\prime }={\bar{a}}_{ij},a_{il}^{\prime }=a_{ij}|A^{\prime },\alpha ,\beta \right)q_{m}\left(A^{\prime },A\right)}{\prod_{k\in \left\{j,l\right\}}\left[\prod_{i,a_{ik}}N\left(z_{i}|h\left({\hat{x}}_{k}\right),Z_{i}\right)\right]p\left(a_{ij}={\bar{a}}_{ij},a_{il}=a_{ij}|A,\alpha ,\beta \right)q_{m}\left(A,A^{\prime }\right)}\right).$$

### 2.8. Sampling Procedure

In this section, we illustrate the sampling algorithm which is composed of all steps presented in the previous sections.

The initiation is given by the generative process of the IBP distribution, which is described in detail in [16]. This generative process is naturally modified so that the conditions (1) are fulfilled (concerning the bistatic space and the proposed targets). The result of this procedure is the association matrix A, the columns of which represent the proposed targets. Target parameters are estimated with regard to the number of associated tracks. In Section 2.2, we denoted $m_{k}$ the number of ones in *k*-th column of the association matrix. In our application, this quantity also represents the number of bistatic tracks assigned to the *k*-th target. If $m_{k}\in \left\{1,2\right\}$ we are unable to initiate the target with full parameters. Therefore, we randomly generate the 6D target position in the area of measurement and find its projection onto the ellipsoid surface ($m_{k}=1$) or onto the ellipse obtained as the curve of two ellipsoid intersections ($m_{k}=2$). This random initiation serves mainly visualisation purposes as the target position is marginalised again once a new measurement is added or some measurement is removed. If $m_{k}\geq 3$ and all of the measurements share a common receiver or transmitter, the initiation is solved using Equations (14) and (15). Otherwise, if $m_{k}\geq 3$ but there is no common focus of the ellipsoids of measurement, the initiation is solved in a more general way, using (13) where the maximisation itself is solved using the log-likelihood and gradient descent algorithms. The same initiation scheme is used through the whole sampling process whenever target position estimation is needed.

The sampling process, given the initial chain state, proceeds as follows. At each iteration, we try to propose one SM move and one R move. Proposal distributions of these moves were described in the previous section together with their respective acceptance probabilities. If the proposal is accepted (i.e., if the realisation of the random variable $U∼\mathrm{Uniform}\;\left(0,1\right)$ is smaller or equal to the ratio $\rho \left(\left(A^{\prime },\theta^{\prime }\right),\left(A,\theta \right)\right)$) the affected columns are modified together with the corresponding target positions. This way, the sampler travels through the parametric space described in Section 2.5. Some of the samples will have different dimensions (i.e., the number of targets) than the others. Note that even if two samples have the same dimension, it does not mean that the target positions have to be the same, since the assignment of tracks to targets can be completely different. For tracking purposes, however, we do not need the whole distribution (described by the obtained set of samples), but rather the “best estimate”, in some sense, is required. Obvious and widely used choices are the maximum aposteriori (MAP) estimate and the maximum likelihood (ML) estimate. In our experiments, the ML estimate was used, which also corresponds to the way in which the target positions are marginalised.

We illustrate the way in which the chain moves between dimensions using one specific chain. The simulation parameters are not important in this case, as we are only concerned with the way the chain moves in the parametric space. The algorithm performance analysis, for which the simulation parameters are important, is provided in the results section of this paper. There were four targets simulated in the area of measurement, three of them with measurements in four bistatic spaces, one of them with only three measurements, and no false measurements were included.

**Figure 4:**
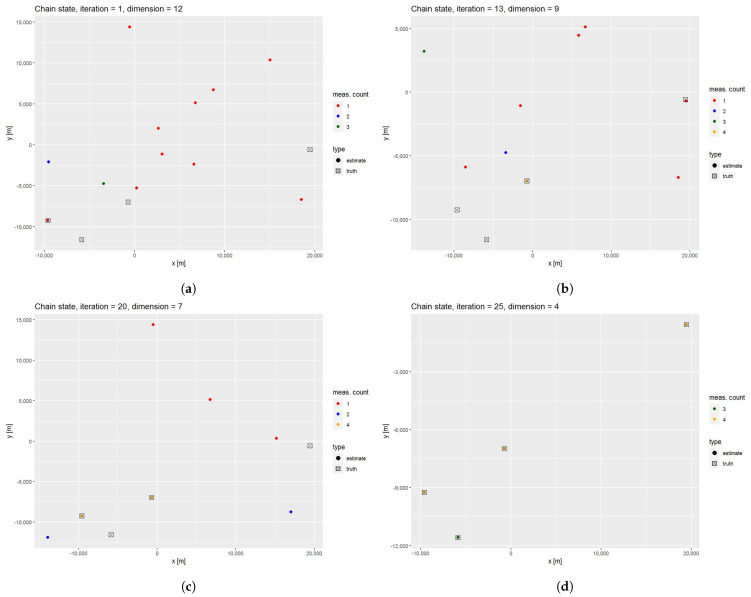
Four different iterations of the chain. At each iteration, we differentiate between estimates with the different number of bistatic measurements (red for one bistatic measurement, blue for two, green for three and yellow for four bistatic measurements). Dot marks are used for estimated positions and boxes for the true target positions: (**a**) chain state at the first iteration with 12 initiated targets, none of the four measurement targets and only one nearby the true target location; (**b**) chain state at the 13th iteration with 9 estimated targets, one of them consisting of four measurements; (**c**) chain state at the 20th iteration with 7 estimated targets, two of them consisting of four measurements, both of them nearby the true target’s location; and (**d**) chain state at the 25th iteration with 4 estimated targets, each corresponding to the true target’s location.

Four different chain states (target positions in the x−y plane) together with the actual target positions are available in Figure 4. Note that even though we illustrated the target states in the x−y plane, the whole computation was performed in the 6D space described in Section 2.5 and the simulated targets also have different *z* coordinates. As we can see, the chain was initiated with highly separated bistatic measurements (10 one measurement targets, 1 two measurement target and 1 three measurement target) and with the initial dimension $n_{1}^{t}=12$. Note that only one of the initial target states corresponds to the actual target position. During the first few iterations, mainly the SM moves were accepted, which led to the creation of the first four measurement targets at iteration 13 and the second one at iteration 20. Both of these targets correspond to the actual targets. In the later iterations, SM and R moves are accepted at approximately the same rate and at iteration 25, all actual target positions were resolved. This illustrates the capability of the sampler to travel through the states with different dimensions. Even though we selected interesting iterations for illustration purposes, the dimension can also grow between iterations. This is solely dependent on the value of the acceptance ratio.

## 3. Results

In this section, we evaluate the performance of our new proposed algorithm. The evaluation was performed using simulated data since these allow us to compare results with the actual truth, which is usually not available when dealing with data from the real system. For the data simulation, we set up an MSPSR system with two receivers and two transmitters, i.e., the system comprises four bistatic spaces. Positions of the sites are available in Table 1. We simulated targets in a local 3D Cartesian space (position and velocity) uniformly in a block-shaped space. The number of simulated targets ranges from 2 to 8. To make the situation closer to reality, all simulated targets were detected and tracked in all bistatic spaces, however, there were also bistatic tracks that did not represent any of the simulated targets. The number of additional false bistatic tracks is always equal to the number of simulated targets. The bistatic space in which those bistatic tracks were simulated was randomly and uniformly chosen. Bistatic positions of the tracks were provided to the association with a diagonal covariance matrix, with the variance of bistatic range $\sigma_{r}^{2}=1000$ m and the variance of bistatic velocity $\sigma_{v}^{2}=4$ m/s. Using the repeated Monte Carlo simulation of data for a different simulated number of targets (and additional false bistatic tracks), we evaluated the number of true-positive (TP) associations, which is the number of associations created by the algorithm which correspond with the actual simulated targets. We also evaluated the number of false-positive (FP) associations, which is the number of associations created by the algorithm, which did not correspond with any of the simulated targets. The last important quantity is the number of false-negative (FN) associations, which is the number of simulated targets which the algorithm did not resolve. These three quantities were evaluated in each Monte Carlo run. The IBP hyperparameters were set to α=0.25 and β=1.9.

### 3.1. Reference Deghosting Algorithms

To make the evaluation more beneficial, we decided to employ two other deghosting algorithms on the same simulated data. The first of the algorithms is a modification of the deghosting algorithm for a single frequency network (SFN) [9]. This algorithm can be easily used for FM-based MSPSR system with the advantage of the absence of transmitter uncertainty. Due to the number of bistatic spaces available (the simulated system comprises four bistatic spaces), we initiated the Cartesian estimates from pairs of bistatic tracks in the chosen *z* coordinate. The clustering procedure was then performed by the likelihood ratio test suggested in [9] with spatial false return density $\rho_{F}=1e^{-11}$ and $p_{D}=0.9$ for all bistatic spaces. In [5], it is suggested to take into account the error caused by replacing the unknown height with a constant. The correction was done in the covariance matrix through the linearisation of the model, which may not work well with different geometries. This in particular may be responsible for quite a high number of false-positive associations (since they could not be merged with their actual neighbours). We refer to this method as the ellipse intersection method (EI method).

The second compared method is loosely based on a similar principle. However, instead of initiating from two measurements and coping with errors caused by unknown height, we initiate targets from all combinations of the three measurements. If there is a target with more than three measurements, the estimates from subsets of three measurements should be relatively close and we can test that using the estimated Cartesian covariance matrices and Mahalanobis distance between the estimates. On the other hand, if there are two triplets from two different targets, they should be far apart from each other. By fusing the close groups and simple fusion rules (the larger the group the better, and the smaller the Mahalanobis distance the better), we can create a feasible association algorithm. We refer to this algorithm as the three-dimensional method (3D method).

### 3.2. Results

As we already mentioned, the performance comparison was performed using simulated data. We evaluated the number of true-positive (TP) associations, the number of false-negative (FN) associations, and the number of false-positive (FP) associations. The TP and FN quantities are complementary and together they sum up the number of simulated targets. The achieved values for all of the three metrics for the new proposed algorithm are available in Table 2.

The displayed values are the average from 100 Monte Carlo simulations of different target positions and bistatic spaces of false measurements, both of which were chosen at random with uniform distribution. As we can see, up to five targets can be clearly resolved, without almost any FP associations and no unresolved target.

The number of unresolved targets stays low even for a higher number of simulated targets, however, due to the increased concentration of false bistatic tracks, the number of FP associations (non-existent targets) grows. This is mainly because in our MSPSR geometry, there are plenty of false intersections of the three ellipsoids. Many of them could be dismissed using kinematic limits and limits for the area of the target position. However, such restrictions were not applied in any of the methods used. Another possible reason for the number of FP associations is the natural inclination of the mixture models to the overestimation of the number of components. Our findings are consistent with the theoretical analysis provided in [34].

**Figure 5:**
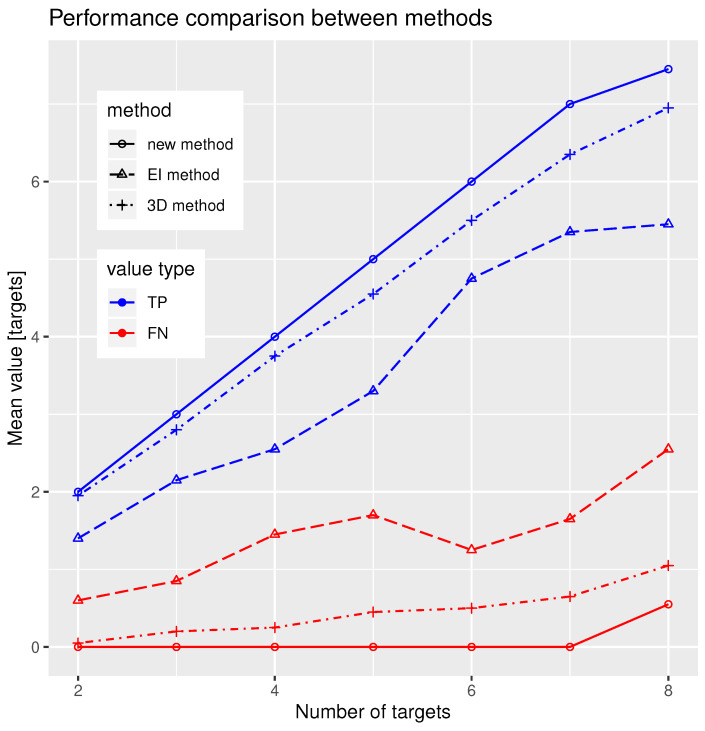
Performance comparison between the three methods. Two quantities, the number of true-positive (TP) resolved targets (blue) and false-negative (FN) unresolved targets (red) are visualised. Solid line together with circular points are used for the new algorithm, dashed line with triangular points for the ellipse intersection (EI) method and dash-dotted line with cross points for the three-dimensional (3D) method.

To compare the performance of our new algorithm, we must run the same simulations for the other two deghosting methods, i.e., the ellipsoid intersection (EI) method and three-dimensional (3D) method, with the same setup and number of Monte Carlo simulations. The comparison of all of the three methods is in Figure 5. Methods are differentiated using line type (new method = solid, EI method = dashed, 3D method = dash-dotted) and TP and FN quantities are differentiated using line colour (TP = blue, FN = red). As we can see, in all cases, the new method outperforms the other algorithms, i.e., the number of successfully resolved targets is always the highest for the new method, while the number of unresolved targets is always the lowest. These results are valid for the simulated geometry and parameters, however, the simulation illustrates the ability of the method to perform the correct association under the given conditions.

### 3.3. Convergence Analysis

In this section, we present the results concerning the convergence of the proposed sampler. The results obtained using the simulated data presented in the previous section suggest a good performance in comparison with the alternative algorithms as well as the ability of the proposed method to associate true targets while avoiding the creation of false ones. However, positive results (even if it would be using data from a real radar system) do not guarantee the correctness of the sampler. By the correctness of the sampler, we usually mean that the stationary distribution of the chain produced by the sampler corresponds to the desired one and whether the chain (of a certain length) may have achieved its stationary distribution. Answering both questions simultaneously is very difficult. In this section, we focus on the latter one, i.e., we analyse whether there is some stationary distribution which is eventually achieved by each chain of a certain length that the sampler produces. There are two different ways to perform such an analysis [35]. The first of them assumes some information about the target density being available, which is then incorporated into the analysis. The second way is purely experimental and is based on running multiple different chains and then analysing the differences between the results achieved by the chains. We chose the second way for incorporating information about the target density would be very difficult. The methods for the analysis of multiple runs of the Markov chain were also adapted for the purposes of transdimensional Monte Carlo methods [36]. However, this modification is not suitable in our case, as it requires us to select a set of parameters which retain the same meaning across all possible models.

As we described in Section 2.5, the target positions, which are continuous variables by nature, are marginalised using maximum-likelihood estimates, which helps the sampler as it does not have to propose the positions at random. This also means that we do not need to include these parameters in the analysis of the chain convergence and we only need to deal with the assignment matrix which defines them completely. Since dealing with the two-dimensional assignment matrix is impractical, we can reduce it to a one-dimensional vector variable. This reduction directly follows from the usage of the IBP prior mentioned in Section 2.2, where the probability distribution is formulated for the whole class of equivalence. This is achieved by transforming the columns of the association matrix into binary numbers, where the first row is interpreted as the most significant bit. By forming the actual integers, for each association matrix $A_{j}^{i}$ at the *j*-th iteration of *i*-th chain, we obtain a vector of integers:

(25) $$\tau_{j}^{i}=\left(\tau_{j1}^{i},\tau_{j2}^{i},\cdots ,\tau_{jK_{j}^{i}}\right)$$

where $K_{j}^{i}$ is the number of columns of the matrix $A_{j}^{i}$. By preserving the conditions (1), we guarantee the numbers obtained in one iteration being unique, i.e., $\tau_{jk}^{i}\neq \tau_{jl}^{i}$ for any k≠l. The analysis of $\tau_{j}^{i}$ between different chains or parts of one particular chain generates two problems. First of all, the variable $\tau_{j}^{i}$ varies in dimension (just as the association matrix does) not only between chains but also between different iterations of one particular chain. The second problem arises from the integral nature of the values in the vector $\tau_{j}^{i}$. For integer values, usual test statistics such as $\hat{R}$ [35] are impractical. An overview of test procedures for Markov chains taking values from a categorical variable is presented in [37]. We used the $\chi^{2}$ test originally proposed in [38] which states the null hypothesis $H_{0}$ that all of the analysed chain segments contain sequences of random samples obtained using a common distribution to all chains in the analysis. The alternative hypothesis $H_{1}$ is that the distributions of single chains are different. Note that even if the single chains achieved a stationary distribution, the test would reject the null hypothesis provided that they are not the same for all chains. The rejection of the null hypothesis therefore either states that the sampler does not provide chains with a stationary distribution or that it is different for each chain. For now, let us reduce the association matrix at each iteration *j* of each chain *i* into the single number $K_{j}^{i}$. This way, the chains consist of scalar discrete variables. Let us have *s* different chain segments and the set of all unique observed target counts $\kappa =\left(\kappa_{1},\kappa_{2},\cdots ,\kappa_{r}\right)$. For each chain *i*, we evaluated $f_{kl}^{i}$ as the number of transitions from dimension $\kappa_{k}$ to dimension $\kappa_{l}$. The test statistic of the null hypothesis was then given by [37]

(26) $$X_{f}^{2}=\sum_{i=1}^{s}\sum_{k=1}^{r}\sum_{l\in R_{k}}\frac{f_{k}^{i}{\left({\hat{p}}_{kl}^{i}-{\hat{p}}_{kl}\right)}^{2}}{{\hat{p}}_{kl}}$$

where:

(27) $$\begin{array}{cc} f_{k}^{i}= & \sum_{l=1}^{r}f_{kl}^{i}\\ {\hat{p}}_{kl}= & \frac{\sum_{i=1}^{s}f_{kl}^{i}}{\sum_{i=1}^{s}f_{k}^{i}}\\ {\hat{p}}_{kl}^{i}= & \frac{f_{kl}^{i}}{f_{k}^{i}}\\ R_{k}= & \left\{k|{\hat{p}}_{kl}>0\right\}. \end{array}$$

According to [38], this test statistic follows the $\chi^{2}$ distribution with the degrees of freedom given by

(28) $$\sum_{k=1}^{r}\left(a_{k}-1\right)\left(b_{k}-1\right)$$

where:

(29) $$\begin{array}{cc} a_{k}= & |\left\{i:f_{k}^{i}>0\right\}|\\ b_{k}= & |\left\{l:{\hat{p}}_{kl}>0\right\}|. \end{array}$$

We generated 10 independent chains from the sampler, using simulated data from the previous section. Each of the chains contained 10,000 samples (i.e., we worked with long runs of the sampler). The analysed segments were then obtained by taking the second half of each chain, i.e., each segment contained 5000 samples. Then, the test statistic (26) was calculated and the value $X_{f}^{2}=8.33$ with n=10 degrees of freedom was obtained. Since the assumed probability distribution of the test statistic was $\chi^{2}$, the value of the cumulative density function is 0.40. Therefore, the p-value is 0.60 which allows us to accept the null hypothesis with the usual significance level α=0.05 and even α=0.1. This test has shown us that the sampler proposed in Section 2 produces chains, which converge to a common stationary distribution concerning the assignment matrix dimension.

**Figure 6:**
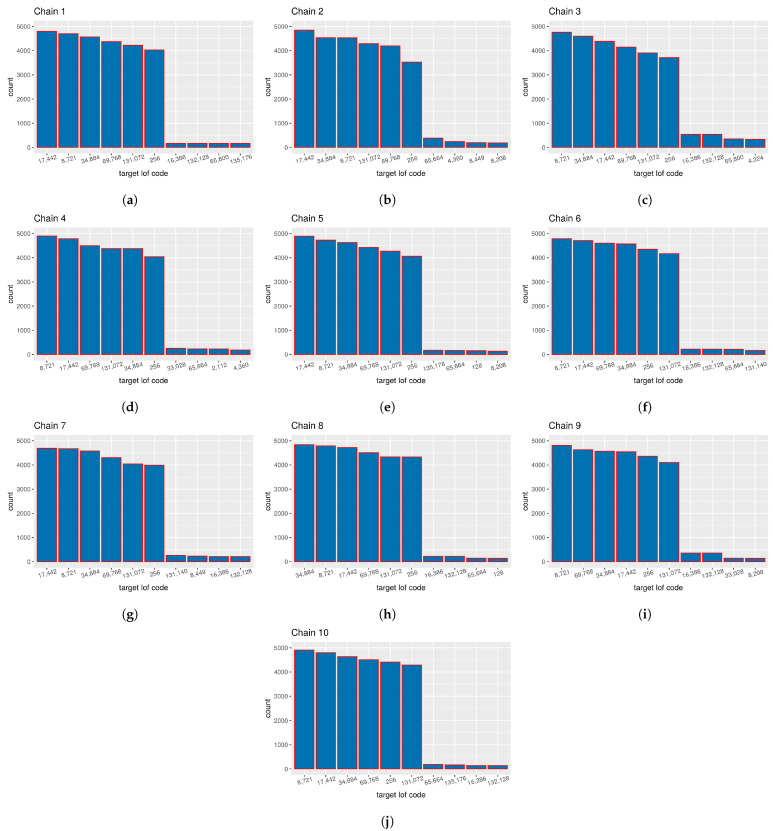
Histogram of top 10 count target lof codes: (**a**) histogram from chain no. 1; (**b**) histogram from chain no. 2; (**c**) histogram from chain no. 3; (**d**) histogram from chain no. 4; (**e**) histogram from chain no. 5; (**f**) histogram from chain no. 6; (**g**) histogram from chain no. 7; (**h**) histogram from chain no. 8; (**i**) histogram from chain no. 9; and (**j**) histogram from chain no. 10.

An appropriate method for the analysis of vectors $\tau_{j}^{i}$ is not known to the authors of this paper. However, once we validate the convergence concerning the number of targets, we can perform the graphical validation of the obtained targets using the histogram of their counts in different chains. Such a histogram is presented in Figure 6. As the chain travels through the parametric space, it discovers multiple different target proposals expressed as columns of the association matrix. Each column can be transformed using the procedure described in Section 2.2, which is used to form classes of equivalence over association matrices. These codes are common even for different runs of the sampler because they are bound to the source data, provided that the ordering of bistatic tracks was not changed. Note that this does not have anything to do with the exchangeability property of the IBP prior, but rather it is a necessary condition for the identification of the specific associated groups of bistatic tracks across the iterations of single chains as well as between multiple chains. We can see that the first six targets in each histogram are the same codes, only with different ordering and counts. These six codes correspond to the true simulated targets and two separated clutter measurements. The rest of the histograms are random targets with rather small counts, which are results of the probabilistic nature of the MCMC method.

**Figure 7:**
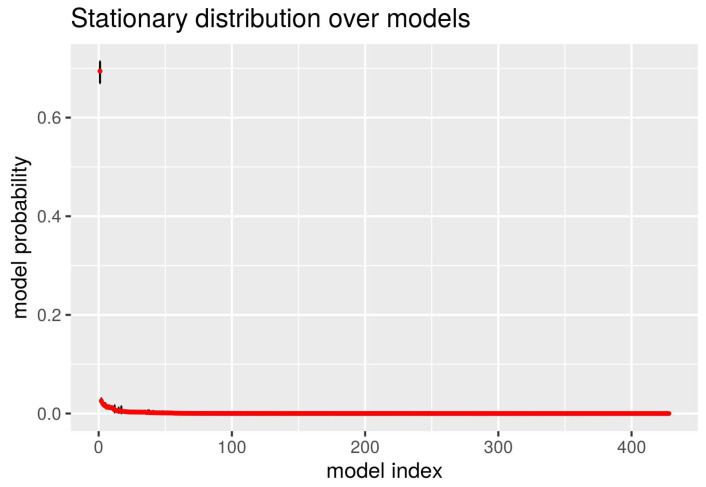
Stationary distribution over the models encountered in the chains produced by the sampler. Red points represent the median and the black bars represent the range between the 5th and 95th percentile. The model labels were assigned again after the posterior evaluation so that they are sorted in descending order. The distinction between the first and the rest of the models is visible and holds even with respect to the precision.

Another interesting question is, once the convergence has been verified, what is the uncertainty of the model posterior probabilities estimated from the chains produced by the sampler. For this purpose, we decided to use the approach developed in [39]. The analysis is based on the estimation of the Markov chain transition matrix where the states represent the current model. In our case, each model is differentiated by its vector of *lof* codes presented in Section 2.2. Note that each model can have a different dimension and therefore the number of codes can be also different. We assigned one numeric label to each unique model and these labels were used in the sampler output. Then, the matrix of frequencies N consisting of entries $n_{ij}$ was built, which represent the number of transitions from a model with label *i* to a model with label *j*. The matrix N is then used as input to the sampled transition matrix $P^{\left(r\right)}$ prior distribution, which was proposed to be:

(30) $$p_{i}^{\left(r\right)}∼D\left(n_{i1}+\epsilon ,n_{i2}+\epsilon ,\cdots ,n_{iI}\right)$$

where $p_{i}^{\left(r\right)}$ denotes the *i*-th row of the sampled transition matrix $P^{\left(r\right)}$, *I* denotes the overall number of models, D denotes the Dirichlet distribution and ϵ is the prior parameter. The prior parameter was set to ϵ=1/I, as it is suggested in [39] for better numerical stability. The samples of the stationary distribution are generated using the normalised eigenvector of $P^{\left(r\right)}$ corresponding to the unit eigenvalue. For the rest of the procedure, please refer to the original paper [39]. The resulting model posterior probabilities, together with the fifth and the ninety-fifth percentiles (vertical black bar) are plotted in Figure 7.

## 4. Discussion

The bistatic track association and deghosting method presented in this paper relies on two techniques. The first of them was the IBP prior distribution for the association matrix and the second one is the RJMCMC inference procedure with a custom set of moves related to the bistatic track association problem.

The suitability of the IBP prior was analysed in Section 2.2. The main argument for its usage is the exchangeability property, which allows us to compose targets and tracks with arbitrary ordering without any influence on the results. The second argument would be the two-parameter version of the distribution. The two parameters can be used to set the expected number of the association matrix columns (i.e., the expected number of targets). This property can also be observed in the results for the simulated data where the number of false-positive targets (i.e., the number of excess columns) is kept relatively low, even for a higher number of tracks. The real-world justification of this approach is the direct connection between the number of targets assumed to be present in the area of measurement and the association scheme (i.e., the association between bistatic and Cartesian spaces). Such a connection is usually missing in the classical deghosting algorithms and we see this as an improvement achieved by the proposed method.

In Section 3, the method assessment using the simulated data was presented. First of, the ability of the proposed method to solve the association problem was tested. This was achieved through the repeated simulation of target measurements and the resolved targets were compared with the known truth. The evaluation of true-positive and false-negative associations suggests a good ability of the method to successfully resolve the actual targets. This is emphasised via a comparison with two alternative algorithms, both of which are outperformed by the proposed method. However, due to the geometrical properties of the bistatic geometry and the nature of the method, the number of false-positive associations grows with the number of actual targets and the number of false measurements. Note, however, that the growth is slower than in the case of the compared algorithms. Since the proposed method is a Markov chain Monte Carlo (MCMC) method, it was considered necessary to assess the convergence of the chains produced by the sampler and to analyse the resulting stationary distribution. This assessment was performed in two steps, both of which used 10 long runs of the sampler with 10,000 iterations (but only the second half of each chain was used in the analysis). In the first part of the analysis, the convergence with respect to the number of resolved targets was performed. For this purpose, the $\chi^{2}$ test for categorical data was utilised and the calculated test statistic verified with a high level of significance that the chains were from a common distribution of the number of targets. The second part of the chain analysis was based on the estimation of the transition matrix between different models (i.e., different association matrices), which is suitable for the transdimensional MCMC. The models with a varying number of parameters were reduced to a single number, the model label. In addition, using the Dirichlet prior for the transition probabilities between models, this analysis allowed us to assess the model posterior probabilities as well as the precision of these probabilities. The graphical results suggest that there is only one model with significant posterior probability and this probability is known with relatively high precision. All of the three analyses verified the good performance of the proposed bistatic track association and deghosting method.

From the modelling perspective, as it was pointed out by the reviewers, the sensitivity of the results with respect to the prior and proposal probability distribution was not analysed. However, as a matter of future work, we plan to provide a comparison of the results using different priors on the association matrix, as this is the key component of our model. However, as far as we know, there are not many probability distributions which do possess the same properties as the two parametric IBP probability distributions. One of the interesting options would be to use the prior used in [11] and compare the results.

The most computationally expensive part is the marginalisation of the target states. At every proposed step, the association of bistatic tracks is changed for a certain subset of targets. For every target influenced by this change in association, we need to compute again the target position in the maximum likelihood sense. The starting point of each such maximisation is computed using the closed form method (14), where the most computationally expensive operation is the multiplication of the coordinate matrix S transposed by itself. The inverse of this product is just an inverse of 3×3 matrix and this inverse is used in the rest of the computation for one target. Only a few iterations are required to correct this initial estimate, if necessary (e.g., if there are measurements which do not fit into the closed form initiation scheme). The number of iterations is also dependent on the precision that is required. We argue that, with respect to the usual targets, if the change in position is less than 0.01 m, the iterations can be stopped. If the maximisation is performed iteratively, the most computationally expensive part is the evaluation of the normal likelihood function, where the exponent corresponds to the Mahalanobis distance. The inversion of the measurement matrix can be solved by precomputation (the data do not change during the inference) and is even easier in the case where we have the same measurement precision for all data. Partial derivatives of the projections from the Cartesian to any of the bistatic spaces can be expressed in closed form. For the rest of the computation, the complexity is comparable to any other classical MCMC method.

The main limitation of the experiments performed in this paper is the bistatic setup. The simulation of the real setup used two transmitters and two receivers. There are systems with many more sites of both kinds, however, they are rarely used in the literature, e.g., [1,2,3,4,5]. In this regard, the setup with more than one receiver and more than one transmitter seems to be more general. The limitation with respect to the number of simulated targets is also present, since the simulation results were only evaluated up to eight simulated targets. However, this is not so uncommon in the tracking literature [1,2] and others. Together with the limitations in the simulation setup design, one of the limitations is the simulation itself. However, the experiments with real data were spared as a matter of future work, since the results’ analysis and data description would take up most of the manuscript. Other limitations such as the fixed choice of the proposal distribution were already mentioned; however, we do believe that the results are nonetheless convincing.

## 5. Conclusions

In this paper, we developed a new bistatic track association and deghosting algorithm. The core of the algorithm is based on the Bayesian approach, namely the hierarchical model which utilises Indian buffet process (IBP) as the prior distribution for the association matrix between the bistatic and Cartesian spaces. The inference of the targets was performed using the reversible jump Markov chain Monte Carlo (RJMCMC), which allowed the method to naturally traverse across association hypotheses with a varying number of targets. In this paper, detailed descriptions of the Bayesian model, the parametric space and the sampler moves are provided. The method assessment was performed using a simulated bistatic setup where the simulated data contained both missing and false measurements. The results show a good performance in comparison with two alternative algorithms. The simulated data were also used to analyse the statistical properties of the chains produced by the sampler in terms of convergence and posterior probabilities. Using statistical testing, the convergence with respect to the number of targets was verified. Further research will be concerned with the sensitivity analysis with respect to the prior probability distribution and assessment of the method using data from a real radar system.

## Figures and Tables

**Table 1:** Cartesian coordinates of four site positions, two receivers (Rx) and two transmitters (Tx).

Site Type	x (m)	y (m)	z (m)
Rx	8311.076	−3793.234	246.0192
Rx	−9001.145	−1833.460	267.4876
Tx	1260.241	5365.893	233.9839
Tx	−1260.366	−9750.959	306.8714

**Table 2:** Association results for the new proposed method. Average numbers of true-positive (TP), false-positive (FP), and false-negative (FN) associations from 100 Monte Carlo simulations are shown.

n. of Targets/Case	TP Assoc.	FP Assoc.	FN Assoc.
2	2.0	0.0	0.0
3	3.0	0.0	0.0
4	4.0	0.4	0.0
5	5.0	0.0	0.0
6	6.0	1.0	0.0
7	7.0	1.2	0.0
8	7.4	2.1	0.6

## Data Availability

Not applicable.
